# HLA-DP diversity is associated with improved response to SARS-Cov-2 vaccine in hematopoietic stem cell transplant recipients

**DOI:** 10.1016/j.isci.2023.106763

**Published:** 2023-04-26

**Authors:** Juliette Villemonteix, Vincent Allain, Emma Verstraete, Debora Jorge-Cordeiro, Gérard Socié, Alienor Xhaard, Cyrille Feray, Sophie Caillat-Zucman

**Affiliations:** 1 Laboratoire d’Immunologie, Hôpital Saint-Louis, Assistance Publique-Hôpitaux de Paris (AP-HP), Université Paris Cité, 75010 Paris, France; 2 INSERM UMR 976, Université Paris Cité, Institut de Recherche Saint-Louis (IRSL), 75010 Paris, France; 3 Service d’hématologie-greffe, Hôpital Saint-Louis, AP-HP, Université Paris Cité, 75010 Paris, France; 4 Centre Hépato-Biliaire, Hôpital Paul-Brousse, AP-HP, Université Paris-Saclay, FHU Hepatinov, 94800 Villejuif, France; 5 Institut National de la santé et de la recherche médicale (INSERM) UMR-S 1193, 94800 Villejuif, France

**Keywords:** Immunology, Virology

## Abstract

Allogeneic hematopoietic stem cell transplantation (allo-HSCT) recipients show lower humoral vaccine responsiveness than immunocompetent individuals. HLA diversity, measured by the HLA evolutionary divergence (HED) metrics, reflects the diversity of the antigenic repertoire presented to T cells, and has been shown to predict response to cancer immunotherapy. We retrospectively investigated the association of HED with humoral response to SARS-CoV-2 vaccine in allo-HSCT recipients. HED was calculated as pairwise genetic distance between alleles at HLA-A, -B, -C, -DRB1, -DQB1, and -DPB1 loci in recipients and their donors. Low anti-spike IgG levels (<30 BAU/mL) were associated with short time from allo-SCT and low donor DPB1-HED, mostly related to donor DPB1 homozygosity. The diversity of donor HLA-DP molecules, assessed by heterozygosity or sequence divergence, may thus impact the efficacy of donor-derived CD4 T cells to sustain vaccine-mediated antibody response in allo-HSCT recipients.

## Highlights

• Hematopoietic stem cell transplant recipients have impaired response to SARS-CoV-2 vaccine
• HLA-DPB1 divergence in the donor is associated with low levels of anti-spike IgG
• Donor HLA-DPB1 homozygosity is associated with no SARS-CoV-2 vaccine response

## Graphical abstract

**Figure:**
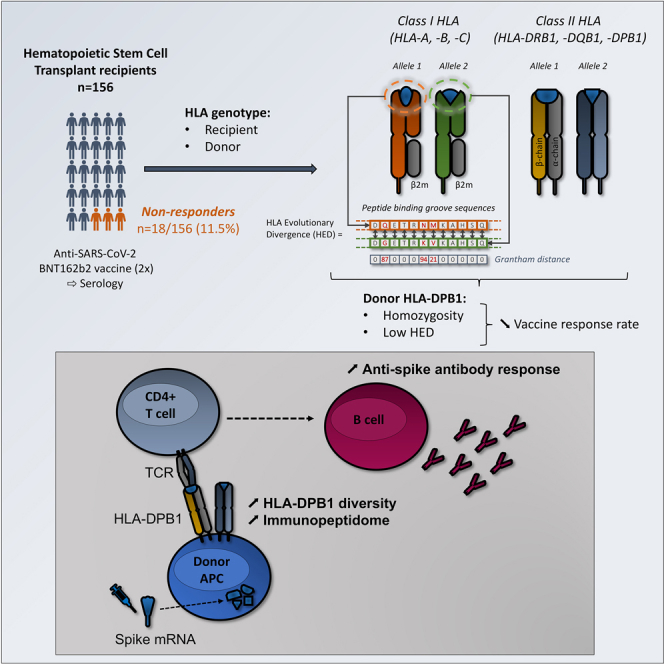

## Teaser

Immunology; Virology **Highlights Hematopoietic stem cell transplant recipients have impaired response to SARS-CoV-2 vaccine HLA-DPB1 divergence in the donor is associated with low levels of anti-spike IgG Donor HLA-DPB1 homozygosity is associated with no SARS-CoV-2 vaccine response**

## Introduction

Allogeneic hematopoietic stem cell transplantation (allo-HSCT) recipients show dampened immune responses to SARS-CoV-2 vaccine,^1^ despite the immunogenicity of this vaccine in the general population. Presentation of vaccine antigens to T lymphocytes is an essential component of vaccine immune response. The highly polymorphic HLA class I (HLA-A, -B, and-C) and class II (HLA-DR, -DQ, and -DP) molecules bind and present vaccine-derived peptides, to CD8 and CD4 T lymphocytes respectively, subsequently initiating antigen-specific immune responses. According to the heterozygote advantage, originally observed in the context of infectious diseases, heterozygous HLA genotypes enable presentation of a wider repertoire of antigenic peptides to T cells, which in turn supports a more diversified T cell response.^2^^,^^3^ The functional difference between two HLA alleles can be further captured by the HLA evolutionary divergence (HED) metric, which quantifies the sequence divergence between the peptide-binding domains of two homologous alleles at a given locus and provides a primary measure of the breadth of the immunopeptidome presented to T cells. We and others have shown that HED is associated with the strength of T cell responses in various clinical contexts, such as response to cancer immunotherapy, autoimmunity, and allogeneic responses.^4^^,^^5^^,^^6^^,^^7^^,^^8^^,^^9^ Herein, we report the impact of HLA diversity on humoral response to SARS-CoV-2 vaccine in allo-HSCT recipients.

## Results

We studied 156 adult allo-HSCT recipients who received two doses of the Pfizer BNT162b2 vaccine 3 to 4 weeks apart between January and July 2021 at a median time of 44 months (range 3–205) after transplantation. Clinical and biological characteristics of the patients are shown in Table 1. No patient was vaccinated earlier than 3 months post-HSCT and none had a clinical history of COVID-19. At the time of vaccination, 71 (45.5%) patients still received graft versus host disease (GVHD) prophylactic or immunosuppressive treatment. Anti-SARS-CoV-2 Spike protein (S) IgG was routinely quantified as binding antibody units (BAU/mL) 30 days after the second dose. Serology at baseline was not available in all patients and was therefore not considered. According to guidances from the Francophone Society of Bone Marrow Transplantation and Cellular Therapy (SFGM-TC), non-responders were defined as anti-S IgG levels <30 BAU/mL, low responders as < 30 but <250 BAU/mL, and strong responders as ≥ 250 BAU/mL (Table 1). Among the 156 patients, anti-S IgG were detectable in 138 (88.5%) patients: 112 patients (71.8%) developed a strong response, 26 (16.7%) were low responders, and 18 (11.5%) were nonresponders.

**Table 1:** Univariate analysis of humoral response to BNT162b2 mRNA vaccine in allo-HSCT recipients

Variable		Response (n = 138)	No response (n = 18)	Total (n = 156)	p value
**Clinical characteristics**					

Age at HSCT (y)	median (range)	49.5 (18.1–71.1)	58.8 (17.4–69.3)	50.8 (17.4–71.1)	0.074
Gender, n (%)	Male	86 (62.3)	13 (72.2)	99 (63.5)	0.575
	Female	52 (37.7)	5 (27.8)	57 (36.5)	
Age at vaccination (y)	median (range)	54.8 (21.7–75.1)	62 (28.3–70.5)	55.3 (21.7–75.1)	0.286
Donor type	Haplo	13 (9.4)	2 (11.1)	15 (9.6)	0.409
	MRD	50 (36.2)	3 (16.7)	53 (34.0)	
	MUD	66 (47.8)	11 (61.1)	77 (49.4)	
	MMUD	9 (6.5)	2 (11.1)	11 (7.1)	
Time from HSCT (months)	median (range)	49.9 (3.3–204.7)	29.1 (4.5–132.3)	44.3 (3.3–204.7)	**0.013**
Disease Risk Index	very high	3 (2.2)	0 (0.0)	3 (1.9)	0.789
	high	26 (18.8)	3 (16.7)	29 (18.6)	
	intermediate	86 (62.3)	11 (61.1)	97 (62.2)	
	low	14 (10.1)	3 (16.7)	17 (10.9)	
	Unknown	9 (6.5)	1 (5.6)	10 (6.4)	
Graft type	PBSC	121 (87.7)	17 (94.4)	138 (88.5)	0.688
	BM	16 (11.6)	1 (5.6)	17 (10.9)	
Donor age (y)	median (range)	34 (15–74)	32 (21–65)	34 (15–74)	0.497
Conditioning	MAC	50 (36.2)	3 (16.7)	53 (34.0)	0.263
	RIC	85 (61.6)	14 (77.8)	99 (63.5)	
	Sequential	2 (1.4)	1 (5.6)	3 (1.9)	
ATG	no	57 (41.3)	5 (27.8)	62 (39.7)	0.495
	yes	80 (58.0)	13 (72.2)	93 (59.6)	
Ongoing GVHD	no	98 (71.0)	12 (66.7)	110 (70.5)	0.684
	yes	40 (29.0)	6 (33.4)	46 (29.5)	
Immunosuppression	yes	61 (44.2)	10 (55.6)	71 (45.5)	0.632
	no	76 (55.1)	8 (44.4)	84 (53.8)	
Steroids	no	113 (81.9)	14 (77.8)	127 (81.4)	0.921
	yes	25 (18.1)	4 (22.2)	29 (18.6)	
Ruxolitinib	no	130 (94.2)	16 (88.9)	146 (93.6)	0.723
	yes	8 (5.8)	2 (11.1)	10 (6.4)	
CsA	no	121 (87.7)	14 (77.8)	135 (86.5)	0.429
	yes	17 (12.3)	4 (22.2)	21 (13.5)	
**HLA Data Donor**					

HED-A	median (range)	6.7 (0–13)	6.9 (0–10.6)	6.7 (0–13)	0.407
HED-B	median (range)	8 (0–14.3)	8.9 (7.0–14.2)	8.1 (0–14.3)	0.059
HED-C	median (range)	5.7 (0–8.2)	7.2 (1.3–8.1)	5.8 (0–8.2)	0.199
HED-DRB1	median (range)	10.6 (0–18.4)	9.6 (0–18.4)	10.5 (0–18.4)	0.497
HED-DQB1	median (range)	10.6 (0–19.2)	10.3 (0–19.2)	10.6 (0–19.2)	0.312
**HED-DPB1**	**median (range)**	**4 (0–10.6)**	**1.4 (0–7.8)**	**3.9 (0–10.6)**	**0.023**
DRB1 homozygosity	yes	11 (8.0)	1 (5.6)	12 (7.7)	1.000
	no	127 (92.0)	17 (94.4)	144 (92.3)	
DQB1 homozygosity	yes	20 (14.5)	5 (27.8)	25 (16.0)	0.269
	no	118 (85.5)	13 (72.2)	131 (84.0)	
**DPB1** homozygosity	**yes**	**28 (20.3)**	**9 (50.0)**	**37 (23.7)**	**0.012**
	no	110 (79.7)	9 (50.0)	119 (76.3)	
DPB1∗04:01	yes	90 (65.2)	12 (66.7)	102 (65.4)	1.000
	no	48 (34.8)	6 (33.3)	54 (34.6)	
**HLA Data Recipient**					

HED-A	median (range)	7 (0–13)	6.5 (0–10.6)	6.6 (0–13)	0.447
HED-B	median (range)	8 (0–14.3)	8.5 (6.3–14.2)	8.1 (0–14.3)	0.114
HED-C	median (range)	5.4 (0–8.2)	7.2 (1.3–8.1)	5.7 (0–8.2)	0.056
HED-DRB1	median (range)	10.4 (0–18.4)	9.2 (0–18.4)	10.3 (0–18.4)	0.574
HED-DQB1	median (range)	11 (0–19.2)	10.8 (0–19.2)	11 (0–19.2)	0.808
HED-DPB1	median (range)	4 (0–10.6)	3.9 (0–10)	4 (0–10.6)	0.584
DPB1 homozygosity	yes	30 (21.7)	5 (27.8)	121 (77.6)	0.564
	no	108 (78.3)	13 (72.2)	35 (22.4)	
DPB1 mismatch	0	75 (54.3)	6 (33.3)	81 (51.9)	0.214
	1	41 (29.7)	7 (38.9)	48 (30.8)	
	2	22 (15.9)	5 (27.8)	27 (17.3)	
**Immune cells (numbers/μL)**					

Lymphocytes	median (range)	1,822 (280 - 5,966)	1,900 (420 - 7,245)	1,822 (280 - 7,245)	0.438
CD4 T cells	median (range)	473 (33 - 1,830)	506 (40–583)	482.5 (33 - 1,830)	0.428
CD8 T cells	median (range)	667 (45 - 3,304)	933 (72 - 1,828)	672.5 (45 - 3,304)	0.507
B cells	median (range)	307 (0–1,656)	26 (0–1,200)	307 (0–1,656)	0.328
NK cells	median (range)	244 (36 - 1,728)	126 (26–436)	236 (26 - 1,728)	0.104

ATG: Anti-ThymoGlobulin; BM: bone marrow; CsA: Ciclosporin A; GVHD: graft-versus-host disease; MAC: myeloablative conditioning; MRD: matched related donor; MUD: matched unrelated donor; MMUD: mismatched unrelated donor; PBSC: peripheral blood stem cells; RIC: reduced intensity conditioning. Significant P values are indicated in bold.

HLA-A, -B, -C, -DRB1, -DQB1, and -DPB1 typing at second field resolution was previously performed in all recipients and their donors. The protein sequence divergence (HED) was calculated for all allele pairs at each locus in both recipients and donors.

**Figure 1:**
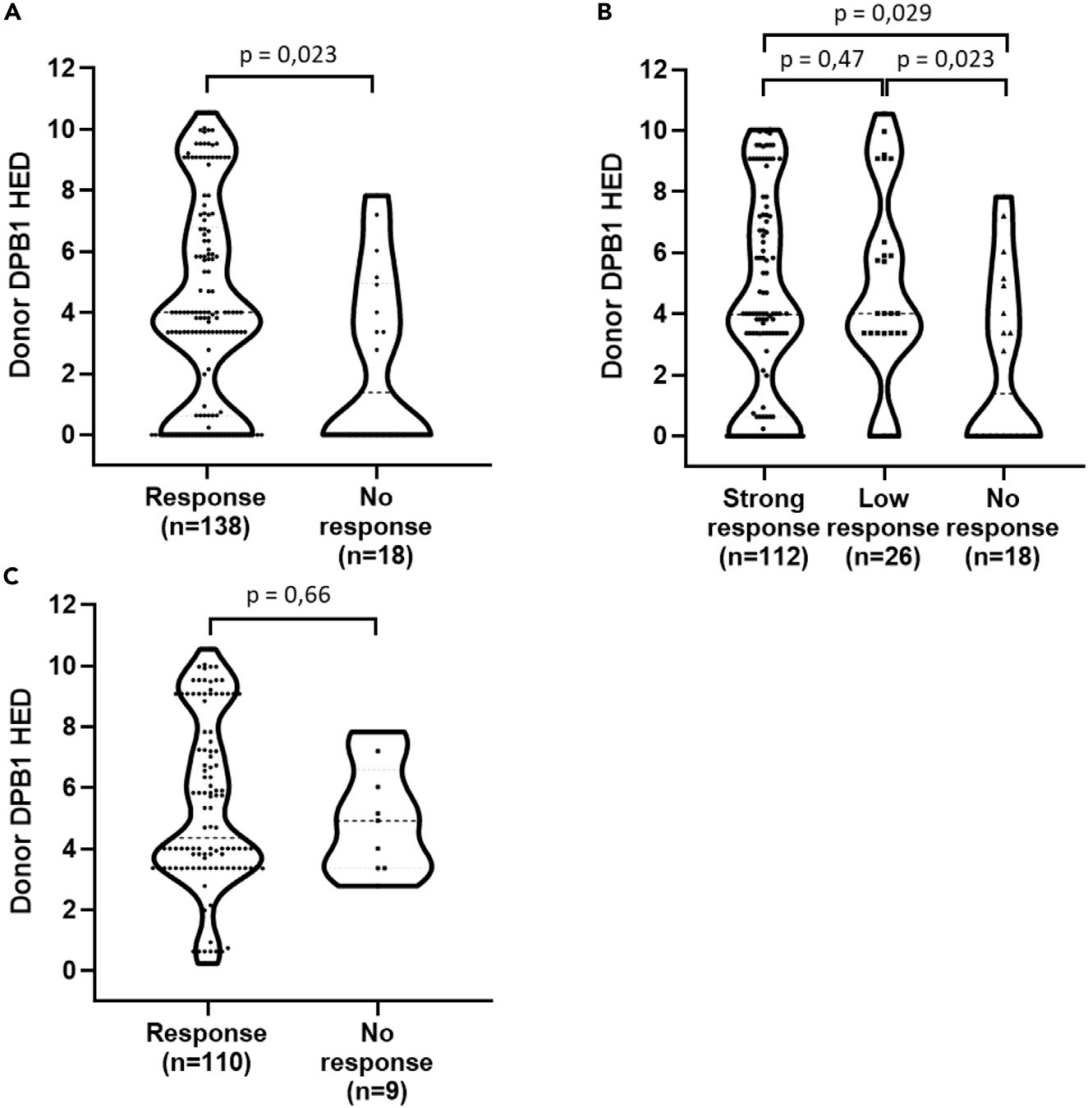
HED at DPB1 locus of the donor and response to BNT162b2 mRNA vaccine in allo-HSCT recipients (A–C) Violin plots with median values are shown according to (A) response or no response; (B) high, moderate or no response; (C) response or no response in DPB1 heterozygous individuals only (n = 119). Two-sided Mann-Whitney P-values.

In univariate analysis comparing responders to nonresponders, shorter time from allo-HSCT and homozygosity or low HED at the DPB1 locus of the donor were associated with no response (Table 1). Donor DPB1 homozygosity was more frequent in nonresponders than in responders (50% versus 20.3%, p = 0.012). Of the 37 recipients of DPB1∗ homozygous donors, 9 were nonresponders compared to only 9 of the 117 recipients of DPB1∗ heterozygous donors (24% and 7.7%, respectively, p = 0.005). Moreover, DPB1-HED was lower in nonresponders than in responders (mean 4.0 versus 1.4, p = 0.023), with no significant difference between low and strong responders (Figure 1). We observed no effect of recipient homozygosity or HED at any locus, or of donor-recipient HLA mismatch, specifically DPB1 mismatch (Table 1). No individual HLA allele of the donor or the recipient was associated with the vaccine response. In particular, the genome-wide significant association of HLA-DQB1∗06 alleles with higher levels of anti-spike IgG at 28 days after the first dose of the ChAdOx1 nCoV-19 vaccine in participants of a large vaccine efficacy trial in the United Kingdom^10^ was not observed in our smallest allo-HSCT cohort (DQB1∗06 present in 34.7% of responders and 38.8% of nonresponders, ns). Moreover, the frequency of DPB1∗04:01, which is among the most common HLA alleles in the general population and could have explained the effect of DPB1 homozygosity or HED, was equally distributed in responders and nonresponders (65.2% and 66.6%, respectively) (Table 1).

Notably, the response rate did not differ by donor type (haplo-identical, mismatched unrelated, or fully matched unrelated or related), immunosuppression status (ongoing treatment with steroids, ruxolitinib, or cyclosporine), presence of active acute or chronic GVHD, or immunological recovery (total, CD8 and CD4 T, B and NK lymphocyte counts) at the time of vaccination (Table 1).

In multivariate logistic analysis, lack of vaccine response was associated with shorter time from allo-SCT (p = 0.033), donor DPB1 homozygosity (p = 0.031) or low donor DPB1-HED (p = 0.010). When excluding the 37 DPB1 homozygous donors, the significant effect of DPB1-HED was lost, suggesting that the lower response rate was mainly related to DPB1 homozygosity.

## Discussion

The development of anti-SARS-CoV-2 immunity is particularly important in allo-SCT recipients, as they have poor outcome after COVID-19 infection. We found that 88.5% of allo-SCT recipients developed an anti-S IgG response after 2 doses of the BNT162b2, a high rate in line with other studies with this vaccine.^11^^,^^12^ SARS-CoV-2 mRNA vaccines have a strong ability to induce CD4 follicular helper T (T_FH_) cell responses which support germinal center formation and differentiation of memory and antibody-secreting B cells.^13^^,^^14^ Therefore, it is not surprising that the diversity of donor HLA class II molecules, assessed by heterozygosity or sequence divergence, may impact the efficacy of donor-derived CD4 T cells to sustain vaccine-mediated antibody response in allo-HSCT recipients. As expected, with the progressive replacement of the recipient’s immune system by a new one derived from donor HSCs, no effect of the recipient’s HLA diversity was observed in our study. Interestingly, the predominant role of HLA-DPB1 relative to -DRB1 and -DQB1 is fully consistent with a recent study highlighting the key role of DPB1-restricted T_FH_ responses against immunodominant spike peptides in establishing a robust and persistent immunity after BNT162b2 vaccine in healthy individuals.^14^ Moreover, recent data point to unique features of HLA-DP molecules that can bind peptides in a reverse orientation, thereby broadening the antigen repertoire recognized by CD4 T cells.^15^

### Limitations of the study

Our study is limited by the relatively small sample size of our cohort, lack of baseline serology in most patients, and antibody titers measured with three different commercial assays. However, it helps in understanding mechanisms of impaired vaccine immune response in immunosuppressed patients. Notably, the 18 nonresponders received a third vaccine dose, of whom 16 had serological evaluation: 4 still failed to mount a serological response and 3 of them (75%) had a DPB1 homozygous donor, compared to 5 of the 12 responders (41.7%). If confirmed in a replication cohort, these data suggest that allo-HSCT recipients of DPB1 homozygous donors should be offered a different platform for a subsequent vaccination.

## STAR★Methods

### Key resources table

**Table:** 

REAGENT or RESOURCE	SOURCE	IDENTIFIER
**Software and algorithms**		

IMGT/HLA database	Robinson et al.^16^	https://www.ebi.ac.uk/ipd/imgt/hla/
R 4.0	R Development Core Team	http://www.R-project.org/

### Resource availability

#### Lead contact

Further information and requests for resources and reagents should be directed to and will be fulfilled by the lead contact, Sophie Caillat-Zucman (sophie.caillat-zucman@aphp.fr).

#### Materials availability

This study did not utilize any physical material.

#### Patient details

We retrospectively studied 156 adult allo-HSCT recipients who received two doses of the Pfizer BNT162b2 vaccine 3 to 4 weeks apart between January and July 2021. HLA-A, -B, -C, -DRB1, -DQB1 and -DPB1 typing at second field (four-digit) resolution was available for all recipients and their donors. All patients gave their consent for data collection before transplantation.

### Method details

#### Anti-spike IgG quantification

Response to SARS-CoV-2 vaccine was defined according to quantitative serological assays using the anti-SARS-CoV-2 S assay (Roche Diagnostics), the SARS-CoV-2 IgG II Quant assay (Abbott Diagnostics) or the SARS-CoV-2 Trimeric S IgG assay (Diasorin). For standardization of the results, the concentrations were transformed using conversion factors (cf.) from the arbitrary units (AU) of each manufacturer to Binding Antibody Units (BAU = cf. x AU), according to the first WHO international standard (NIBSC code 20/136).

#### HLA evolutionary divergence (HED) calculation

The respective protein sequences of the peptide-binding groove (exons 2 and 3 for HLA-A, -B, and -C, and exon 2 for HLA-DRB1, DQB1 and -DPB1) were extracted from the IMGT/HLA database.^17^ For each of the 6 genes, the protein sequence divergence between 2 alleles (HED) was computed for all possible combinations of allele pairs encountered in the cohort. The calculation of HED between aligned allele pairs at a given locus was measured as a continuous metric using the Grantham distance,^16^ which takes into account the differences in the composition, polarity and volume of between two amino acids. HED is null in case of homozygosity.

### Quantification and statistical analysis

Standard descriptive statistics were used to summarize baseline demographic, clinical and biological characteristics. The outcome was the vaccinal response defined as an ordinal categorial variable: no response, moderate response, and strong response. Ordered logistic regression was used for the univariable and multivariable analysis of relevant covariates. All analyses were performed with R 4.0 using the “Grantham” and “MASS” packages. The threshold for significance was p value <0.05.

Associations of HLA allelic frequencies with the vaccinal response were evaluated using a two-sided Fisher’s exact test with the Bonferroni correction.

## Data Availability

This paper does not report original code. Any additional information required to reanalyze the data reported in this paper is available from the lead contact upon reasonable request.
